# Water wavenumber calibration for visible light optical coherence tomography

**DOI:** 10.1117/1.JBO.25.9.090501

**Published:** 2020-09-15

**Authors:** Tingwei Zhang, Aaron M. Kho, Vivek J. Srinivasan

**Affiliations:** a University of California Davis, Department of Biomedical Engineering, Davis, California, United States; b University of California Davis, School of Medicine, Department of Ophthalmology and Vision Science, Sacramento, California, United States

**Keywords:** optical coherence tomography, retinal imaging, dispersion compensation, wavenumber calibration

## Abstract

**Significance**: Visible light optical coherence tomography (OCT) is emerging for spectroscopic and ultrahigh resolution imaging, but challenges remain. Depth-dependent dispersion limits retinal image quality and current correction approaches are cumbersome. Inconsistent group refractive indices during image reconstruction also limit reproducibility.

**Aim:** To introduce and evaluate water wavenumber calibration (WWC), which corrects depth-dependent dispersion and provides an accurate depth axis in water.

**Approach:** Enabled by a visible light OCT spectrometer configuration with a 3- to 4-dB sensitivity roll-off over 1 mm in air across a 90-nm bandwidth, we determine the spectral phase of a 1-mm water cell, an affine function of water wavenumber. Via WWC, we reconstruct visible light OCT human retinal images with 1.3-μm depth resolution in water.

**Results:** Images clearly reveal Bruch’s membrane, inner plexiform layer lamination, and a thin nerve fiber layer in the temporal parafovea. WWC halves the processing time, while achieving the same image definition as an assumption-free gold standard approach, suggesting that water wavenumber is a suitable proxy for tissue wavenumber. WWC also provides a depth axis in water without explicitly assuming a group refractive index.

**Conclusions:** WWC is a simple method that helps to realize the full potential of visible light OCT.

Optical coherence tomography (OCT)^1^ measures “echo time” delay of light, or optical group delay of light, or optical group delay, τ, to reconstruct the reflectivity of an object as a function of image depth, $z_{\mathrm{img}}$. Neglecting aliasing effects, image depth is determined by $z_{\mathrm{img}}=v_{g,\mathrm{img}}\tau /2$, where $v_{g,\mathrm{img}}$ is the group velocity used to reconstruct the image, and the factor of 2 arises from the double pass reflection geometry. Typically, $v_{g,\mathrm{img}}=c/n_{g,\mathrm{img}}$ is assumed, where c is the speed of light in free space (here used interchangeably with air) and $n_{g,\mathrm{img}}$ is an assumed group refractive index for image reconstruction.

While the assumption of a single, constant group refractive index is reasonable for most OCT imaging systems in the conventional wavelength range from 700 to 1400 nm, recent work has pushed OCT imaging outside of this range toward both shorter^2^^–^^8^ and longer^9^^–^^11^ wavelengths. For large imaging depths, broad bandwidths, and wavelength ranges with high dispersion, differences in group velocity across the spectral bandwidth may lead to depth-dependent dispersion, which degrades image resolution if a constant group index is assumed in reconstruction. Common physical matching and numerical spectral phase correction methods^12^ can only correct dispersion at a single depth and do not address depth-dependent dispersion. Recently, depth-dependent dispersion was identified as a major factor that degrades visible light OCT of the retina,^13^ even for relatively modest bandwidths. A closely related issue is the determination of the group refractive index for image reconstruction. Since water group refractive index varies by a few percent from 400 to 700 nm, axial image dimensions could also vary as different parts of the visible spectrum are utilized if group index variations are neglected.

In spectral/Fourier domain OCT,^14^ depth-dependent dispersion is closely linked with spectral calibration.^12^ Spectral calibration is a process to ensure that interferograms are sampled uniformly in the Fourier conjugate of the variable of interest. For example, if τ is the variable of interest, uniform samples in ω (angular optical frequency) are desired. In spectral/Fourier domain OCT, spectral calibration usually requires a resampling procedure. Notably, Tumlinson et al.^15^ demonstrated correction of depth-dependent dispersion in a glass medium, but not in biological tissue, by programming a tunable laser to sample uniformly in glass wavenumber. Existing approaches for compensation of depth-dependent biological tissue dispersion in spectral/Fourier domain OCT require an additional resampling step after spectral calibration,^13^^,^^16^ complicating image reconstruction.

In this paper, we propose an approach to address group refractive index variations for spectral/Fourier domain OCT in biological tissue. We first perform a series of measurements on a water cell in transmission to determine its spectral phase. We then use this spectral phase to uniformly sample the spectral interference pattern in water wavenumber, implicitly correcting depth-dependent dispersion [Fig. 1(a)]. This contrasts with the conventional approach, which samples uniformly in air wavenumber.^12^^,^^17^^–^^20^ If the water cell dimensions are precisely known, our approach also provides a depth axis for the reconstructed image without requiring assumptions about the group refractive index. We demonstrate our approach in visible light spectral/Fourier domain OCT.

**Fig. 1:**
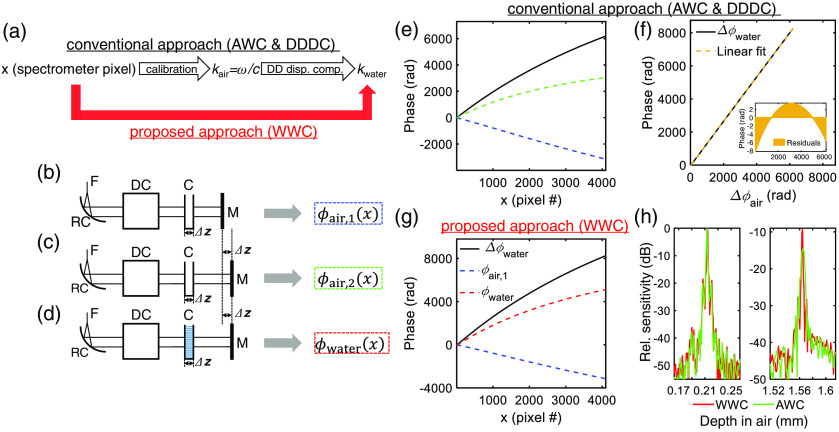
WWC simplifies the transition from uniform x sampling to uniform $k_{\text{water}}$ sampling in Fourier domain OCT. (a) The conventional AWC approach requires resampling followed by DDDC, if required, while the WWC approach implicitly achieves DDDC. (b)–(d) Reference arm configurations for generating spectral phases for the different processing methods [F, single mode fiber; RC, reflective collimator; DC, dispersion compensation components; C, cuvette; M, mirror; Δz, physical distance in Eqs. (1) and (2)]. Note that the cuvette has been filled with distilled water in (d). (e)–(g) Experimental measurements with Δz=1  mm. (e) Conventional AWC method. The blue and green dotted lines show the spectral phases for (b) and (c), respectively. In the conventional AWC approach, DDDC is also required (f) to eliminate residual phase errors (inset). (g) Proposed WWC approach. The blue and red dotted lines show the spectral phases for (b) and (d), respectively. (h) PSFs retrieved by WWC (red) and AWC (green) processing, without [left, corresponding to (b)] and with [right, corresponding to (d)] 1 mm of water.

A spectrometer disperses wavelengths across sensor pixels, represented by the variable x. Conventional spectral calibration uniformly samples the spectral phase corresponding to a known air gap.^17^^–^^20^ This yields uniform sampling in free space wavenumber, $k_{\text{air}}=\omega /c$, which is proportional to the Fourier conjugate of $z_{\text{air}}=c\tau /2$. Therefore, this approach samples uniformly in ω. To achieve uniform sampling in $k_{\text{air}}$, we require a function that represents an affine transformation of $k_{\text{air}}$. Samples that are uniformly spaced in this affine function would also be uniformly spaced in $k_{\text{air}}$. To achieve this, a common approach^17^^–^^20^ is to measure two interferograms and calculate the difference in their spectral phases. The pair of interferograms is acquired sequentially with a path length difference of 2Δz in air, which is achieved by translating the reference arm by Δz [Figs. 1(b) and 1(c)]. The spectral phase difference is given as

$$\Delta \phi_{\text{air}}(x)=\phi_{\text{air},2}(x)-\phi_{\text{air},1}(x)=2k_{\text{air}}(x)\Delta z+\Delta \phi_{\text{air},d}.$$ (1)

Note that a phase shift $\Delta \phi_{\text{air},d}$ has been included to account for possible system drift between the sequential measurements. Since Eq. (1) represents an affine transformation of $k_{\text{air}}$, uniformly sampling this function yields uniform samples in $k_{\text{air}}$ and hence ω.

While ω is invariant, wavenumber is medium specific. In the Fourier domain, depth-dependent dispersion arises from a mismatch between the wavenumber used for calibration and the wavenumber of the medium.^15^^,^^21^ Assuming water is the medium for retinal imaging, the fundamental variable of interest is $z_{\text{water}}$, depth in the specimen, whose Fourier conjugate is proportional to $k_{\text{water}}$. Thus, spectral calibration should perform uniform sampling in $k_{\text{water}}$. To achieve this, we require a function that represents an affine transformation of $k_{\text{water}}$. One such function is the spectral phase of a water slab. To determine this, we again employ a pair of interferograms. This time, the second interferogram is acquired after filling a cuvette of physical length Δz with water and also increasing the reference path by 2Δz in air. For this pair [Figs. 1(b) and 1(d)], the spectral phase difference is given as

$$\Delta \phi_{\text{water}}(x)=\phi_{\text{water}}(x)-\phi_{\text{air},1}(x)=2k_{\text{water}}(x)\Delta z+\Delta \phi_{\text{water},d}.$$ (2)

Again, a phase shift $\Delta \phi_{\text{water},d}$ has been included to account for possible system drift between the sequential measurements. Using this function to achieve uniformly spaced samples in $k_{\text{water}}$, we can compensate for spectrometer and depth-dependent water dispersion in a single resampling step. Also, since Δz is known, the depth axis is automatically calibrated in water without explicitly assuming a group refractive index, as is customary in OCT.

To implement our water wavenumber calibration (WWC) method [Figs. 1(e)–1(h)], a visible light OCT spectrometer^22^ and interferometer were employed. The spectrometer consisted of a reflective collimator (7-mm focal length, RC02APC-F01, Thorlabs, Inc.), diffraction grating (1800  lines/mm, Wasatch Photonics), focusing lens pair (two 250-mm achromats, AC508-250-A, Thorlabs, Inc.), and a complementary metal-oxide semiconductor line scan camera (4096 10  μm×20  μm pixels, SPL 4096-140 km, Basler AG). Instead of the conventional back focal plane configuration,^23^ the alignment was improved by shifting the diffraction grating 46.4 mm from the back focal plane, as shown in Fig. 2(a), to improve the spot size along the horizontal axis at the sensor. The root-mean-squared (rms) spot sizes, based on Gaussian quadrature ray tracing in OpticStudio (Zemax. LLC) [Fig. 2(b)] with a 9.5-mm pupil, support the improved performance of the new configuration. Experimentally, compared with previous configurations,^22^^,^^23^ the point spread function (PSF) roll off improved to 3.4 dB from 6.0 dB over the first millimeter in air. The rolloff of the envelope of the spectral interference pattern was assessed next. Importantly, the rolloff of the new configuration ranged between 3 and 4 dB over the first millimeter in air, from 520 to 610 nm [Figs. 2(c) and 2(d)], and was significantly better than the old configuration at the edges of the spectrum. This improvement proved critical, enabling us to accurately measure the spectral phase of a water cell across our broad visible light OCT bandwidth.

**Fig. 2:**
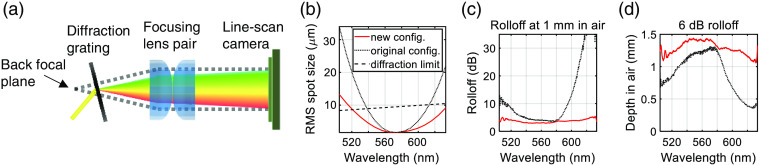
(a) Schematic of spectrometer configuration with diffraction grating shifted from of the back focal plane of the focusing lens pair. (b) Zemax simulations of the rms spot size, based on ray tracing with a 9.5-mm pupil, for both the original back focal plane configuration and the new, shifted configuration suggest improved performance. The diffraction limit is estimated assuming a 3.5-μm mode field diameter at 515 nm and a constant numerical aperture across the wavelength. (c), (d) Experimentally, the rolloff of the spectral interference envelope in the new configuration is significantly improved across the broad bandwidth, enabling accurate measurement of the spectral phase of a 1-mm water cell [Figs. 1(e)–1(g)].

Using this system, we next implemented our proposed WWC approach using a Δz=1  mm cuvette (#EW-83301-10, Cole-Parmer Instrument Co) in the reference arm [Figs. 1(e)–1(h)]. The oscillating part of the interference spectrum was isolated by subtracting the sample arm spectrum and reference arm spectrum, followed by background correction. To determine the spectral phase, we employed a Hilbert transform with phase unwrapping. In Fig. 1(h), the PSFs recovered by air wavenumber calibration (AWC) and WWC are compared without [Fig. 1(b)] and with [Fig. 1(d)] 1 mm of water, optimizing depth-independent dispersion compensation for the PSF without water. As expected, both methods perform well without water [Fig. 1(h), left]. However, as shown in the right panel of Fig. 1(h), WWC recovers near optimal relative sensitivity (−9.1 versus −9  dB predicted from the envelope) and axial resolution (1.91 versus 1.84  μm in air predicted from the envelope) with 1 mm water, whereas AWC does not (−14.6  dB and 3.91  μm for the relative sensitivity and axial resolution in air, respectively).

An additional benefit of our method is the direct calibration of the depth axis in water without assuming a group index. To achieve this, we noted that the phase evolution of $\Delta \phi_{\text{water}}$ across the sensor was 8252 rad [Fig. 1(g)], corresponding to 1313 cycles. Therefore, point (or pixel) 1313 in the discrete Fourier transform corresponds to $z_{\text{water}}=1\;\mathrm{mm}$. Thus, the full depth range (2048 pixels) is 1.56 mm in water, corresponding to a pixel spacing of 0.76  μm in water.

The human retina was imaged *in vivo* with a fiber-based visible light spectral/Fourier-domain OCT system,^22^^,^^23^ incorporating the improved spectrometer in Fig. 2. A retinal image of a healthy 27-year old male (Fig. 3) was acquired with 120-μW incident power at the cornea. This image was generated by averaging a volumetric dataset acquired at a 70 kHz line rate with 1044 A-lines per B-scan over a 6.25 mm fast-axis range, and 200 B-scans over a 0.2-mm slow-axis range for speckle reduction. Transverse and axial motion correction were applied prior to intensity averaging. Raw fringes for images in Fig. 3 were processed with one of three different wavenumber calibration methods: WWC (red), AWC and depth-dependent dispersion compensation (DDDC)^13^ (blue), and AWC (green). The prior assumption-free approach,^13^ which optimized DDDC by minimizing wavelength-dependent image shifts across depth, was taken as the gold standard. Not including the time required for optimization, this approach required two resampling steps,^13^ while the WWC approach employed just one resampling step and did not require an optimization procedure. All methods included transverse-dependent dispersion compensation (TDDC)^13^ [Figs. 3(a)–3(c)]. Zoomed images of the inner retina [Figs. 3(d)–3(f)] demonstrate inner plexiform layer (IPL) lamination. They also show that the inner limiting membrane (ILM) specular reflection is more confined in depth near the foveal pit [Figs. 3(g)–3(i)] by WWC and AWC and DDDC methods than by the AWC method. This spatial confinement of the ILM enables better visualization of the extremely thin nerve fiber layer (NFL) in the temporal retina [Figs. 3(j) and 3(k)], which is confounded with sidelobes by the AWC method [Fig. 3(l)]. The transversely averaged and normalized intensity of the flattened ILM confirms the importance of DDDC, which enables a 20% narrower FWHM, as shown in Fig. 3(m) (Note that the measured intensity FWHM is larger than the intensity FWHM of 0.9  μm predicted from the squared axial PSF, possibly due to the imperfect flattening of ILM prior to averaging). These seemingly subtle improvements are significant when measuring thicknesses of layers at the micron-scale with visible light OCT.

**Fig. 3:**
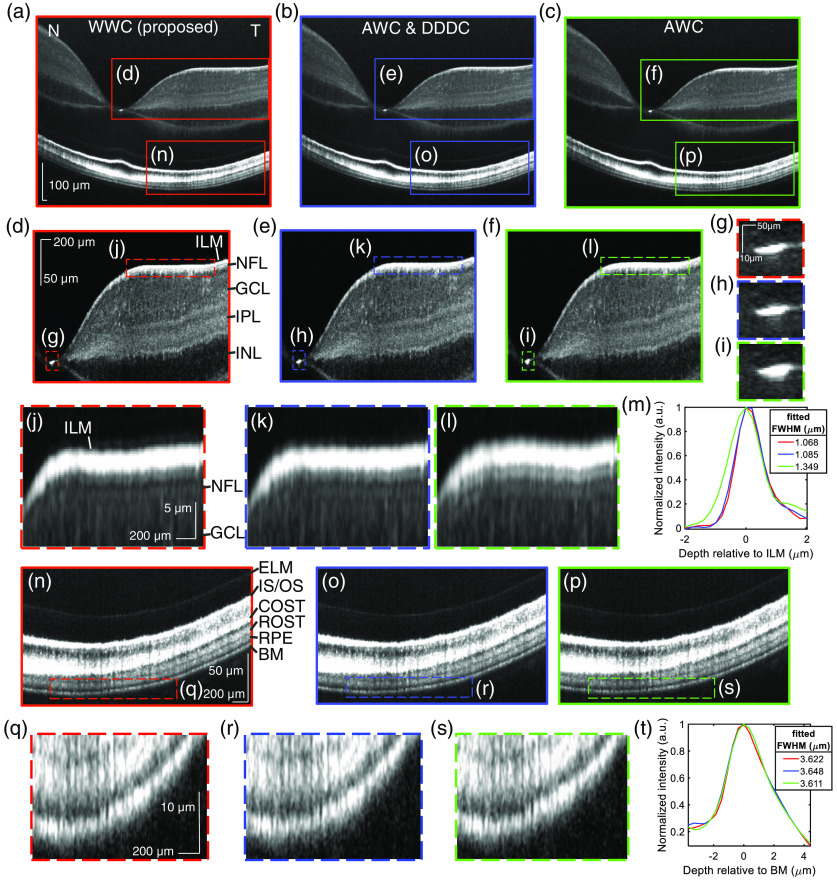
Comparison of visible light OCT human retinal images with different calibration methods. The red boxes or curves represent images from the WWC method. The blue boxes or curves represent the images from previously introduced AWC and DDDC methods.^13^ The green boxes or curves represent the images from conventional AWC method.^24^ TDDC^13^ was performed on all images. (a)–(c) Ultrahigh-resolution cross-sectional retinal images. 200 frames were acquired with the spectrometer shown in Fig. 2 and averaged. N, nasal side of retina; T, temporal side of retina. (d)–(f) Inner retinal zooms on a square root scale, showing IPL lamination. (ILM, inner limiting membrane; NFL, nerve fiber layer; GCL, ganglion cell layer; IPL, inner plexiform layer; INL, inner nuclear layer). (g)–(i) Zooms of the foveal pit. WWC and AWC and DDDC methods show narrower specular reflections than the AWC method. (j)–(l) Zooms of the ILM show that WWC and AWC and DDDC methods reveal details of the NFL below the specular ILM reflection in the temporal retina, which are confounded with sidelobes with the AWC method. (m) The normalized intensity near the ILM and Gaussian fitted FWHMs confirm improved image resolution. (n)–(p) Outer retinal zooms on a log scale. (ELM, external limiting membrane; IS/OS, inner segment/outer segment junction; COST, cone outer segment tips; ROST, rod outer segment tips; RPE, retinal pigment epithelium; BM, Bruch’s membrane). (q)–(s) Image resolution and fitted FWHMs (t) of BM are comparable among the different methods, because TDDC optimization was weighted toward the outer retina.

Zoomed images of the outer retina [Figs. 3(n)–3(p)] visualize the major photoreceptor layers, the retinal pigment epithelium (RPE), and Bruch’s membrane (BM). Zooms of the outer retina and BM [Figs. 3(q)–3(s)], as well as normalized intensity profiles of BM [Fig. 3(t)] suggest that image resolution is comparable for all methods. Indeed, all methods yielded a similar external limiting membrane (ELM) width of 1.02 to 1.06  μm (not shown in figure). This observation can be explained by the fact that TDDC was optimized based on correlation of sub-band images of the entire retina, and that the correlation function is more weighted toward the outer retina, which has a higher intensity than the inner retina. However, our TDDC method was depth independent and could only optimize dispersion at a single depth. Thus, though all methods optimize outer retinal image quality, DDDC must still be achieved to optimize image resolution in the inner retina [Fig. 3(m)].

It is reasonable to question whether water approximates the dispersion of ocular media. While the vitreous humor is predominantly water, the retina contains a significant fraction of other molecules with different refractive indices. In spite of this concern, WWC experimentally achieves similar performance to the empirical DDDC approach (Fig. 3). Better performance than the assumption-free DDDC approach is not expected.

In summary, our practical WWC approach unifies spectral calibration and DDDC into a single step and just requires an additional cuvette. For aqueous tissues, WWC greatly simplifies image reconstruction, while providing a quantitative depth scale in water. We recommend that WWC be considered and implemented on all spectral/Fourier-domain OCT systems, especially those that operate in the visible light range, to ensure optimal performance and reproducibility. The integration of WWC with axial eye motion correction will be the subject of a future investigation.
